# Characterization of a Magnesium Fluoride Conversion Coating on Mg-2Y-1Mn-1Zn Screws for Biomedical Applications

**DOI:** 10.3390/ma15228245

**Published:** 2022-11-20

**Authors:** Sofia Gambaro, M. Lucia Nascimento, Masoud Shekargoftar, Samira Ravanbakhsh, Vinicius Sales, Carlo Paternoster, Marco Bartosch, Frank Witte, Diego Mantovani

**Affiliations:** 1 National Research Council, Institute of Condensed Matter Chemistry and Technologies for Energy, CNR-ICMATE, 16149 Genoa, Italy; 2 Biotrics Bioimplants AG, Ullsteinstrasse 108, 12109 Berlin, Germany; 3 Prosthodontics, Geriatric Dentistry and Craniomandibular Disorders, Charité Universitätsmedizin Berlin, Aßmannshauser Straße 4–6, 14197 Berlin, Germany; 4 Laboratory for Biomaterials and Bioengineering (CRC-I), Department of Min-Met-Materials Engineering and University Hospital Research Center, Regenerative Medicine, Laval University, Quebec City, QC G1V 0A6, Canada

**Keywords:** biodegradable implants, Mg-based screws, MgF_2_ conversion coating, electrochemical impedance spectroscopy, corrosion rate

## Abstract

MgF_2_-coated screws made of a Mg-2Y-1Mn-1Zn alloy, called NOVAMag^®^ fixation screws (*biotrics bioimplants AG)*, were tested in vitro for potential applications as biodegradable implants, and showed a controlled corrosion rate compared to non-coated screws. While previous studies regarding coated Mg-alloys have been carried out on flat sample surfaces, the present work focused on functional materials and final biomedical products. The substrates under study had a complex 3D geometry and a nearly cylindrical-shaped shaft. The corrosion rate of the samples was investigated using an electrochemical setup, especially adjusted to evaluate these types of samples, and thus, helped to improve an already patented coating process. A MgF_2_/MgO coating in the µm-range was characterized for the first time using complementary techniques. The coated screws revealed a smoother surface than the non-coated ones. Although the cross-section analysis revealed some fissures in the coating structure, the electrochemical studies using Hanks’ salt solution demonstrated the effective role of MgF_2_ in retarding the alloy degradation during the initial stages of corrosion up to 24 h. The values of polarization resistance (Rp) of the coated samples extrapolated from the Nyquist plots were significantly higher than those of the non-coated samples, and impedance increased significantly over time. After 1200 s exposure, the Rp values were 1323 ± 144 Ω.cm^2^ for the coated samples and 1036 ± 198 Ω.cm^2^ for the non-coated samples, thus confirming a significant decrease in the degradation rate due to the MgF_2_ layer. The corrosion rates varied from 0.49 mm/y, at the beginning of the experiment, to 0.26 mm/y after 1200 s, and decreased further to 0.01 mm/y after 24 h. These results demonstrated the effectiveness of the applied MgF_2_ film in slowing down the corrosion of the bulk material, allowing the magnesium-alloy screws to be competitive as dental and orthopedic solutions for the biodegradable implants market.

## 1. Introduction

Magnesium-based alloys represent one of the most promising classes of biodegradable biomaterials to produce devices for both orthopedic and dental applications, providing appropriate temporary support while forming new bone [1]. Conventional metals in biomaterial applications, such as stainless steel, Ti-based alloys, and Nitinol, used to produce “permanent” implants, have superior mechanical properties than magnesium-based materials [2]. However, such metallic materials can give rise to allergic reactions, permanent physical irritation, chronic inflammatory local responses [3], and, most importantly, the need to be removed by a follow-up surgery after healing [1]. Magnesium-implants, on the other hand, can naturally degrade in a physiological environment without most of the aforementioned risks [1,4], representing a great alternative to Ti-implants when temporary support or bone regeneration are required. Magnesium is non-toxic and is even an essential element in the human body: it is involved in protein synthesis, activation of various enzymes, and regulation of activities in the neuromuscular and central nervous systems [1,5]. Magnesium also has advantages over other absorbable metals, such as iron and zinc, due to its recommended daily intake in adults (240–420 mg·day^−1^), which is around 50 times that of iron (8–18 mg·day^−1^) and zinc (8–11 mg·day^−1^) [6]. From the perspective of bone implantation, magnesium has the advantage of a Young’s modulus value (41–45 GPa) close to that of natural bone (3–20 GPa) [7] and excellent osteoconductivity [8]. Moreover, Mg ions have been shown to enhance the local release of a neuropeptide (CGRP) from sensory nerve endings in the periosteum. This stimulates the persisting periosteal stem cells to create cortical bone [9], and enhances the Wnt signaling pathway in hBMCs to stimulate cancellous bone growth [10]. In addition, the simultaneous change of oxygen concentration adjacent to corroding Mg implants and the local high Mg ion concentration promotes angiogenesis [11]. The major drawback of magnesium application resides in its fast and uncontrolled dissolution when immersed in physiological media containing chloride; pure magnesium (Mg = 99.9 wt.%; Fe < 40 ppm) has a high corrosion rate of 2.89 mm·year^−1^ in 0.9% NaCl solution [12]. The rapid degradation rate results in mechanical integrity loss [13,14] and hydrogen bubbles formation [15], which can delay bone healing [16], block blood flow, and significantly alter the local pH [17].

To control the magnesium degradation rate and improve its mechanical properties, the addition of alloying elements, forming solid solutions and secondary phases, or the use of special processing or surface modification routes is required [1,18,19]. One of the most common solutions to decrease the corrosion rate is to modify the material surface, a usually simpler and more practical approach than changing the bulk structure and composition [20,21]. Different types of coatings have already been developed for the application of magnesium-alloys, such as β-tricalcium phosphate [22], hydrogenated amorphous silicon [23], nanomembranes obtained by electrospinning [24], electrodeposited hydroxyapatite [25,26], calcium phosphate conversion [27], and magnesium fluoride [28], among others. These coatings can partially control material degradation. In particular, fluoride films have been shown to have higher resistance and lower capacitance than calcium phosphates, and were significantly more effective in delaying the degradation rate of the bulk [29,30]. In addition, fluorine is essential in the human diet and is required for normal dental and skeletal growth; it promotes osteoblast proliferation and increases new mineral deposition in cancellous bone naturally degrading in the human body [29]. The safety of MgF_2_ coatings has already been proven, considering its in vivo solubility and decomposition rate, as well as the fluoride average daily intake in the human diet [31]. Moreover, fluoride-coated magnesium-alloys showed a satisfying biocompatibility toward co-cultivated human smooth muscle and endothelial cells [32]. An extensive in vitro study on the biocompatibility of fluoride-surface-modified AZ31 Mg-alloy revealed a significant stimulation of cell proliferation and a strong enhancement of the mitochondrial respiratory activity when mouse osteoblasts (MC3T3-E1), fibroblasts (L929), and macrophages (J774) cell lines were cultured with the modified alloy [33]. Moreover, the positive effect of MgF_2_ coating in delaying the corrosion of the bulk material to a reasonable level was demonstrated in 2010; electrochemical and immersion tests showed an improved corrosion resistance of fluoride-coated AZ31B alloys in a simulated body compared to the bare Mg-alloy [34]. In 2013, this result was further confirmed and consolidated by experimental tests on a MgF_2_ layer, produced as a conversion coating in an HF bath, which showed an enhancement of the coated material corrosion resistance by increasing the acid concentration [35]. In the same year, it was found that a denser and more uniform MgF_2_ coating was formed on Mg-0.5Ca alloy surfaces by immersion in 40% HF compared to 35% HF-treated samples. Electrochemical results of those samples, obtained in Kokubo’s solution, revealed that the corrosion resistance of fluoride-coated samples was 30 times higher than the untreated ones [36]. An in vivo corrosion study on magnesium alloy LAE442 demonstrated the good corrosion protection of the fluoride layer after implantation of coated and non-coated cylinders into the medial femoral condyle of adult rabbits [37]. The MgF_2_ coating was no longer detected 4 weeks after implantation, and no subcutaneous gas cavities were found in any animals, proving a lower corrosion rate for the coated sample, as well as an adequate host response [37]. More recent studies, conducted in the past five years, have mainly focused on the biological characterization of the coating than on its impact on corrosion resistance. However, the reported findings confirm the positive impact of MgF_2_ towards decreasing corrosion of the bulk material [38], its biocompatibility [38,39,40], and its effect on promoting new bone formation [38,41]. They have also shown beneficial effects in terms of osteogenic differentiation of MC3T3-E1 cells and the vascularization of human umbilical vein endothelial cells (HUVECs) [42]. Table 1 summarizes the conclusions and the outcomes of the cited studies.

Most studies reported in the literature consisted of simple-shape, flat samples. In this work, however, a 3D and complex-shape material was coated with a MgF_2_ layer and studied. The aim was to evaluate the efficiency of the coating process in covering the screw in its entirety, and in decreasing the degradation of the bulk material.

In the present work, a MgF_2_ conversion coating was applied through a wet-chemical process on commercial Mg alloys, WZM211 or Mg-2Y-1Zn-1Mn, developed and patented by *biotrics bioimplants AG*. The purpose of the surface modification technique was to control the corrosion rate of biodegradable dental devices (screws) used in clinical procedures, known as Guided Bone Regeneration (GBR) [43,44]. Degradation of WZM211-coated alloys was investigated during in vitro and in vivo tests. Results revealed that the screw was slowly and completely resorbed after 52 weeks, thus providing adequate fixation in the early critical healing phase [37]. The magnesium fixation screw demonstrated all the key properties required for an ideal temporary fixation screw of membranes used in GBR surgeries [44,45]. Therefore, a characterization study of these materials and their degradation mechanism could allow the tuning of coating properties, making the screws more versatile in terms of performance. For this reason, a deep material characterization including SEM-EDS, AFM, and XPS analyses was proposed. In particular, the corrosion behavior of non-coated and coated screws was evaluated and compared in vitro in artificial physiological fluid using electrochemical techniques. The biological evaluation of these screws has been already carried out by *biotrics bioimplants AG* in a previous work [44].

## 2. Materials and Methods

### 2.1. Materials and Coating Process

NOVAMag^®^ fixation screws (length L = ~13.5 mm; thread diameter Ø = ~1.2 mm), produced by *biotrics bioimplants AG*, were used as substrate materials. The nominal composition of the alloy, named WZM211, is reported in Table 2.

MgF_2_ conversion coating uses the principle of magnesium dissolution and interaction of its ions with fluoride ions, leading to the precipitation of MgF_2_ on the surface of the substrate, while hydrogen evolution takes place. The coating can be produced by immersion, the major safety concern in the use and disposal of fluoride-containing baths. The screws were first cleaned in an isopropanol bath for 20 min and then dried; they were then immersed in a 40% HF solution maintained at room temperature (T = 25 °C) and agitated. After this period of immersion, screws were extracted with a plastic sieve, cleaned in isopropanol, and then dried again. More details about this immersion process are reported in the *botiss biomaterials AG* patents [46]; work on the biological response of these treated Mg-alloys has been recently published [44]. Machined and treated WZM211 screws are here referred to as non-coated and MgF_2_-coated samples, respectively.

### 2.2. Samples Characterization

**Figure 1:**
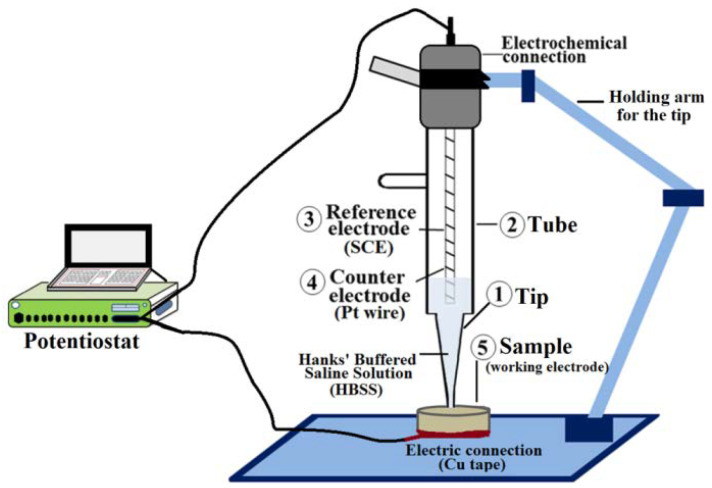
Schematic representation of the mini-cell system adopted in this work.

The morphology and homogeneity of coated and non-coated screw surfaces were evaluated by SEM-EDS (FEI Quanta250 SEM system (Thermo-Fisher, OR, USA)). The cross-section composition of the coated screws was further characterized using a focused ion beam and EDX (model 8530F). The acceleration voltage used was in the range of 10–20 kV. The topography and roughness of the surface were investigated by Atomic Force Microscopy (AFM, Dimension™ 3100, Digital Instrument, CA, USA) in tapping mode with an etched silicon tip (model NCHV, tip radius = 10 nm, Bruker). The chemical composition in the uppermost 5–10 nm of the surface was quantitatively investigated using X-ray photoelectron spectroscopy (XPS) (PHI 5600-ci, Physical Electronics, Chanhassen, MN, USA); the analyses were carried out using an incident angle of 45° with respect to the normal angle of the surface, and a residual pressure of 3 × 10^−9^ Torr. A survey spectrum, covering the range of 0–1400 eV, was first recorded using a standard achromatic aluminum K_α_ X-ray source (1488.6 eV) at 200 W. High-resolution spectra of C1s, O1s, Mg2s, and F1s regions were recorded with a magnesium K_α_ X-ray source (1253.6 eV) at 150 W. No charge neutralization was required. Curves fitting was determined by means of the least-squares method using PHI MultiPak^TM^ software and Gaussian-Lorentz (30–70) functions with a Shirley background. The in vitro corrosion testing of the coatings using electrochemical experiments was conducted using a three-electrode mini-cell system, *MCS* (Ibendorf & Co. GmbH, Bernau bei Berlin, Germany), that tests complex surfaces without any additional preparation, and which has been previously used to study Mg-alloys [47]. A scheme of the *MCS* setup is shown in Figure 1.

The setup was composed of: (1) a plastic tip filled with the testing solution and in contact with the sample surface; (2) a tube, containing (3) a saturated calomel (SCE) as a reference electrode, and (4) a platinum counter-electrode; (5) a working electrode, which is the sample surface to be analyzed. Electric contact was achieved using a conductive copper adhesive stripe on the sample backside and a clamp, to ensure a good contact among the components. A digital microscope (Keyence VHX 900, Keyence Deutschland GmbH, Neu-Isenburg, Germany) was used, with a magnification 200×, to determine the area of the corrosion test spot. The tip and the tube of *MCS* were filled with 3 mL of Hanks’ buffered saline solution (HBSS, Thermofisher product number 14175095). The experiments were performed at room temperature (25 ± 1 °C). The tip of the MCS was placed directly on the surface of the specimen, analyzing 0.4 mm^2^ of its surface in each test; the electrochemical measurements started as soon as the tip had contact with the surface. The experiments were driven by a potentiostat (Autolab PGStat 302, Metrohm AG, Herisau, Switzerland), with frequency response analysis (FRA) software (Nova 2.0) connected to the electrochemical setup. Electrochemical impedance spectroscopy (EIS) tests were performed between 100 kHz and 10 mHz, at a potential amplitude of 10 mV around the OCP. The OCP was registered during the initial 20 s for stabilization, before starting EIS. Measurements were performed at 20 and 1200 s exposure of the surface to the medium. The EIS measurements are only possible on such a short time scale due to the small dimensions of the tip, in the range of 700 µm diameter. In order to investigate if the coating was stable upon prolonged exposure, single EIS experiments were performed up to a period of 24 h. After finishing the test, the sample was dried using pressured air and stored in controlled environmental conditions. Based on ASTM G102, the corrosion rate was defined by:

(1) $$\mathrm{CR}={\;K}_{1}\frac{i_{\mathrm{corr}}\;.W}{n\;.\;\rho \;}$$

where CR is the degradation rate (mm/yr); K_1_ = 3.27 × 10^−3^ mm·g·µA^−1^·cm^−1^·yr^−1^ is a conversion factor; i_corr_ represents the corrosion current density (A); ρ stands for the density of the metal (g/cm^3^); W and n refer to the atomic weight (g) of the element and its valence, respectively. To determine the corrosion current density, the Stern approximation was used, an alternative approach to calculate this parameter from electrochemical data [48]:

(2) $$i_{\mathrm{corr}}=\frac{R\;.\;T}{n.\;F.\mathrm{Rp}\;}$$

where R is the universal gas constant (8314 J/mol K), T represents the temperature (K), and Rp the polarization resistance (Ω.cm^2^).

The polarization resistance (Rp) was determined directly from the EIS curves at the limit where the imaginary part of the impedance reaches zero, as reported in the literature [49]; but in this case, only using the high-medium range frequencies, as the signal response in such a fast-changing surface is not linear in the low-frequency region [50]. The limitation of this approach is that the Rp values determined are only valid within the timescale of the experiment. However, it allows an analysis of reactive samples in a relatively short time frame (5–10 min), in a setup with a small sample area, such as the MCS. This obtains reproducible results. Validation of the results was made by fitting using an RC circuits model (ZView^®^ for Windows, Scribner Associates Inc, Southern Pines, NC, USA).

## 3. Results and Discussion

### 3.1. Coating Morphology and Chemical Composition

**Figure 2:**
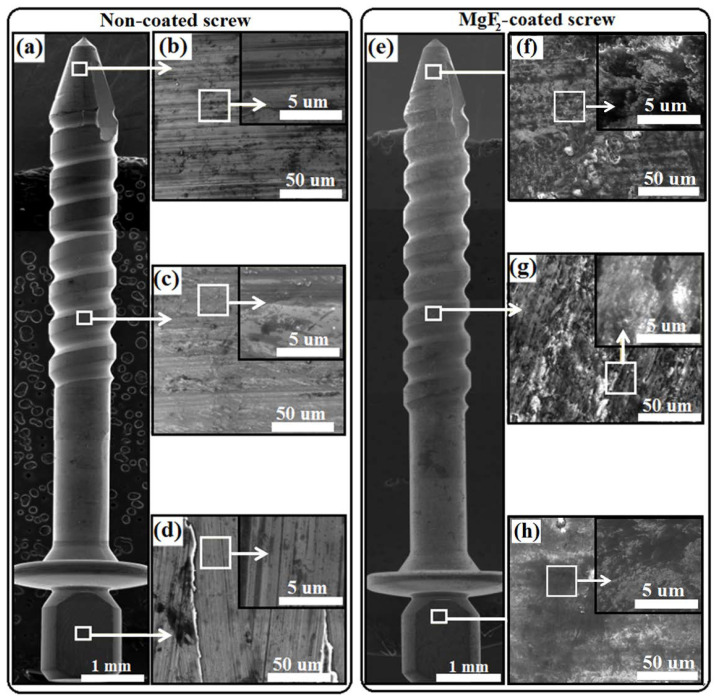
SEM analysis of non-coated (**a**) and MgF_2_-coated screws (**e**); high magnification micrographs of the tip (**b**,**f**), thread (**c**,**g**), and head (**d**,**h**).

Three different parts of the screw, consisting of the tip, thread, and flat head, were considered to evaluate the morphology and elemental distribution of the conversion coating over the entire material surface (see Figure 2 for further details). For non-coated screws, slightly different morphologies were found at the tip and head compared to the thread (Figure 2a–d). The latter showed, in fact, a finer structure with a minor amount of surface lines coming from the machining process. The machining lines and different morphologies could be associated to the parameters used in machining [51]. These different morphologies partially affected the final morphology of the HF-treated samples. It could be observed that a compact film, with the aspect of an amorphous layer, grew on the entire surface of treated samples (Figure 2e–h).

**Figure 3:**
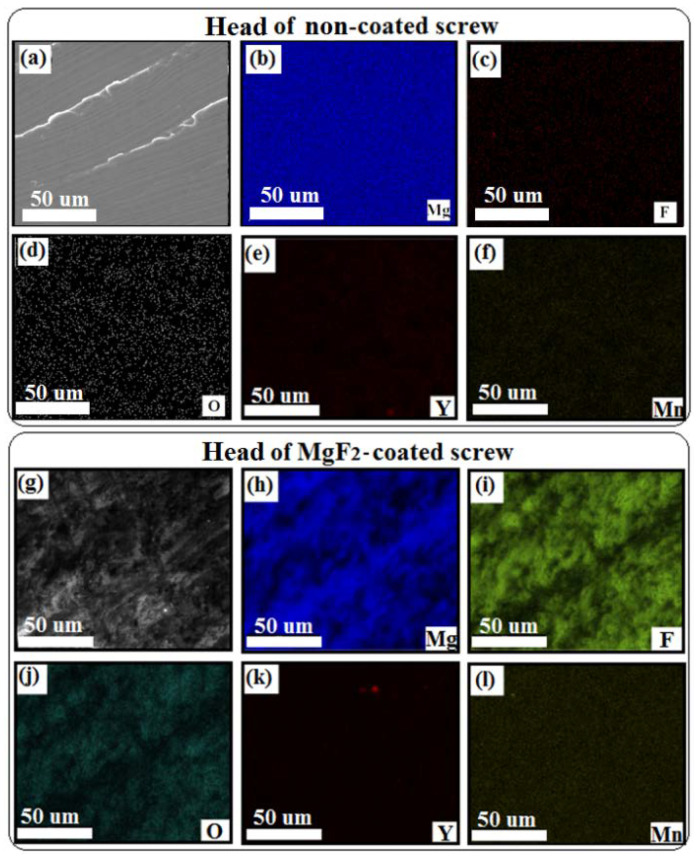
EDS mapping of MgF_2_-coated screws; elements distribution of the tip (**a**–**f**) and of the thread (**g**–**l**).

**Figure 4:**
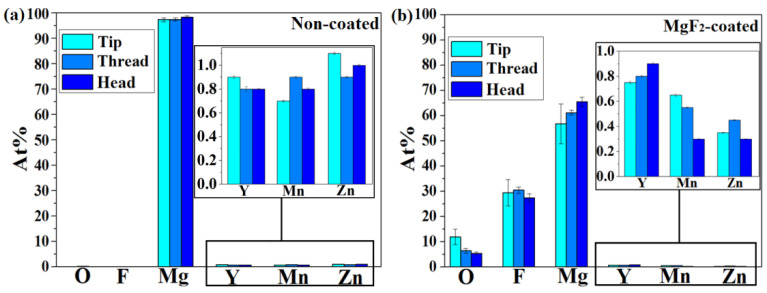
EDS analyses of areas in Figure 2 for non-coated (**a**) and MgF_2_-coated samples (**b**).

**Figure 5:**
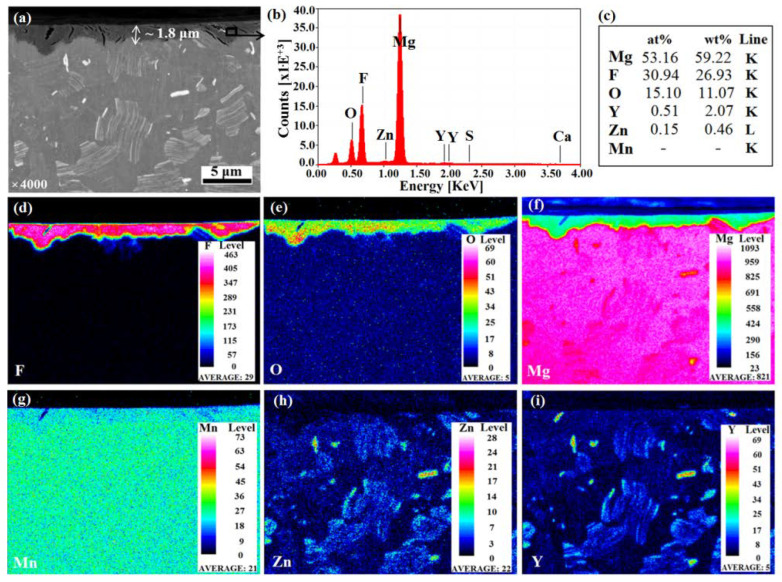
SEM analysis of a cross-sectioned MgF_2_-coated sample (**a**); WDX spectrum (**b**) and elemental compositions (**c**) of the coating (black square of (**a**)); maps of elemental distribution (**d**–**i**). This analysis was performed at ZeLMI, TU Berlin.

The mapping EDS analyses, as shown in Figure 3, revealed the presence of a mixed MgF_2_/MgO layer formed on the entire screw after immersion in HF. In the case of untreated screws, Mg was clearly visible after mapping (Figure 3b) and the amounts of Zn and Mn were close to nominal (1 wt.%), while the Y amount was lower and was always around 1 wt.% (Figure 4). This can be attributed to the fact that Y is not soluble in Mg, and it tends to accumulate and form precipitates, sometimes with Zn, so that it is concentrated in specific places in the alloy. The tip, thread, and head of the screws showed the same elemental distribution; therefore, only the results related to the screw head were selected. EDS elemental maps revealed a homogenous distribution of Mg, O, and F over the entire MgF_2_-coated screw surface; the alloying elements (Y, Zn and Mn) were not found at the sample surface as second phase particles due to their low amount and their distribution in solid solution inside the material bulk (Figure 5).

These elements did not segregate or form second phase compounds, visible in the micrometer range, on the surface of the coated screws. Few isolated particles of Y were found on coated samples (Figure 3e,k) [52]. EDS analysis conducted on coated surfaces confirmed the formation of a mixed MgO/MgF_2_ layer due to the presence of a significant amount of O and F (Figure 4).

The FIB and EDX analyses, conducted on the cross-section of coated samples, showed the presence of a thin interface zone between the substrate and the coating, without relevant diffusion. The coating thickness is not completely homogeneous, ranging from 1.8 to 2.4 µm (Figure 5a), filling the valleys of the surface, resulting in a smoother final surface. EDS spectrum and the related elemental compositions (Figure 5b,c) allowed to confirm the formation of the aforementioned mixed MgO/MgF_2_. 

**Figure 6:**
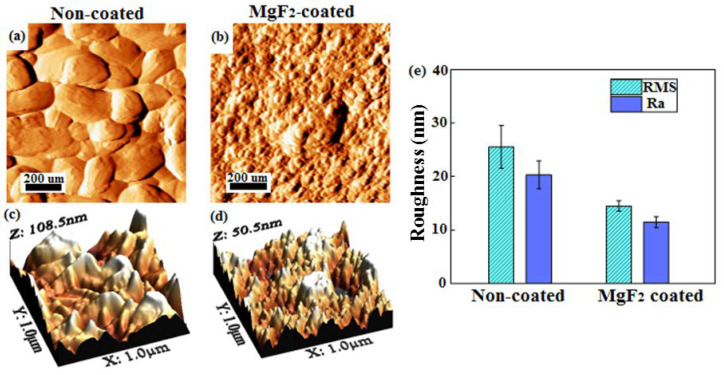
AFM analysis performed on screws head: 2D images of non-coated (**a**) and coated (**b**) screws; 3D-images of non-coated (**c**) and coated (**d**) screws; roughness measurements (**e**).

Moreover, some fissures were visible in the coating cross-section; they had mainly a vertical development that was perpendicular to the surface and to the substrate-coating interface. They were distributed all along the coating and some of them showed a length equal to the coating thickness. The distribution of the alloying elements over the cross-section confirmed the absence of Mn and Zn from the coating, while a small presence of Y particles (Figure 5i) was present. Mn was homogeneously distributed all over the alloy bulk (Figure 5g). Zn and Y were grouped in lamellar structures, dispersed in the alloy bulk. They formed some intermetallic compounds [53], visible in Figure 5h,i, and they were not affected by the process. Roughness measurements and topographical characterization were carried out on both non-coated and MgF_2_-coated screws (Figure 6). The roughness values (RMS and R_a_) of non-coated screws appeared significantly higher than those of coated screws: RMS = 26.5 ± 4.2 nm and R_a_ = 14.3 ± 1.2 nm, respectively (Figure 6e). In addition, the topography of the samples changed as a result of the conversion coating treatment. The topography of coated screws, being smoother than that of non-coated screws, showed a regular texture, exhibiting the formation of grooves with a similar dimension and were homogeneously distributed (Figure 6a–d), as also reported by Makkar et al. [54].

**Figure 7:**
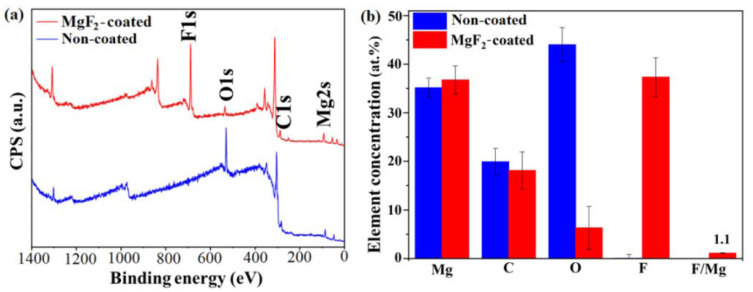
Survey spectra (**a**) and concentration of elements (at%), as well as F/Mg ratio (**b**), of non-coated (blue) and coated surfaces (red).

XPS measurements were also performed to better evaluate and compare the chemical composition of both non-coated and MgF_2_-coated surfaces. Survey spectra of both surfaces, as well as their atomic concentrations, are reported in Figure 7.

**Figure 8:**
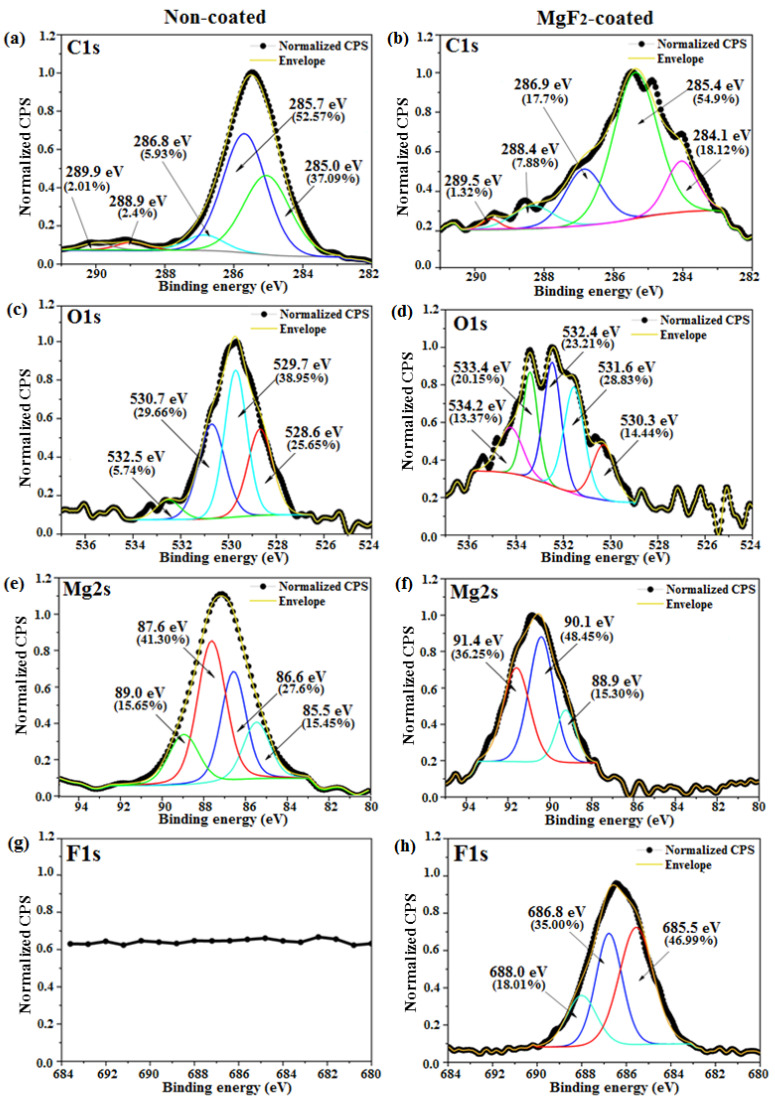
High-resolution spectra of C1s (**a**,**b**), O1s (**c**,**d**), Mg2s (**e**,**f**), and F1s (**g**,**h**) of non-coated and coated samples, respectively.

The surface concentrations of Mg before and after the coating were approximately similar: ~35.1 at. % and ~36.7 at. %, respectively. As expected, coated samples showed a high amount of fluorine (37.3 at. %), while it was totally absent for non-coated samples (Figure 7b). The determined atomic concentrations of O1s on the non-coated and coated surfaces were ~44.1 at. % and ~6.3 at. %, respectively. From the surveys, it was not possible to confirm the presence of the previously mentioned MgO/MgF_2_ layer, as O, for example, it is often found for different kinds of Mg alloys [55,56,57]; Wan et al. found ~51.2 at. % of O on the surface of bare Mg alloy [55]. High-resolution spectra showed that surface chemical species were specific for the two conditions (Figure 8).

In particular, for the coated condition, the peaks of Mg2s, F1s, and O1s were shifted towards higher binding energy that could be related to a variation of the oxidation states on the surface (Figure 8). The deconvoluted C1s spectrum of coated samples showed a peak at ~ 284.1 eV, corresponding to the C-F bonds [58]. The O1s peaks revealed the presence of several species (Figure 8c,d). In particular, a variation was found between the two studied conditions; the binding energies of O1s peaks for non-coated and coated samples were ~529.7 eV and 531.9 eV, respectively. This shifted peak could be related not only to a change in the oxidation state, but also to the bonding between C and F atoms, which highly increased after the coating process. These shifted peaks resulted in the different binding energy for each component. The fit of the Mg2s peak of coated samples revealed the presence of three components at 88.9 eV (MgO), 90.1 eV (Mg(OH)_2_), and 91.4 eV (MgCO_3_). However, as indicated in Figure 8e,f, the location of each component differed from non-coated to coated samples. The high-resolution Mg2s spectra showed that non-coated samples possessed an additional peak located at a binding energy of ~89.0 eV, which was attributed to MgH_2_ [59]. Mg presents a strong tendency to bond with environmental molecules, and this results in the formation of hydroxides and carbonates on the surface [60]. As expected, the high-resolution F1s peaks corresponding to the non-coated condition showed no signal of fluorine. The fitted F1s peak for coated samples showed three peaks located at ~685.5 eV (MgF_2_), ~686.8 eV (C-F bonds), and ~688.0 eV (carbon fluorinated chains) [61]. The different characterization techniques used to evaluate the coating revealed the presence of a mixed MgO/MgF_2_ layer formed on the screw after immersion in HF, even if the F/Mg ratio detected by XPS analysis was sub-estimated (about 1.1). It was then proven that the treatment with HF led to a pronounced magnesium fluoride layer on the screws and significantly reduced the organic contamination, indicated by lower C and O contents on the surface. The formation of surface pores and the mixed nature of the coating could be explained by considering the micro-galvanic cells formed on the sample surface in contact with HF. In the micro-anode region, the magnesium could be dissolved as follows:

(3) $$Mg_{\left(s\right)}\to Mg_{\left(aq\right)}^{2+}+2e^{-}$$

Where the following cathodic reaction occurred:

(4) $$2H_{\left(aq\right)}^{+}+2e^{-}\to \;H_{2\left(g\right)}$$

Meanwhile, the metallic cations coming from the anodic reaction react with the fluoride anions of the solution, forming magnesium fluoride (5), and an oxidation reaction also occurred (6):

(5) $$Mg_{\left(aq\right)}^{2+}+2F_{\left(aq\right)}^{-}\to \;MgF_{2\left(s\right)}$$

(6) $$Mg_{\left(s\right)}+H_{2}O_{\left(l\right)}\to \;Mg{\left(OH\right)}_{2\left(s\right)}+H_{2\left(g\right)}$$

Since Mg(OH)_2_ was not stable in acidic solution [36], it forms other MgF_2_ and MgO:

(7) $$Mg{\left(OH\right)}_{2\left(s\right)}+2HF_{\left(aq\right)}\to \;MgF_{2\left(s\right)}+2H_{2}O_{\left(l\right)}$$

(8) $$Mg{\left(OH\right)}_{2\left(s\right)}\to \;MgO_{\left(s\right)}+H_{2}O_{\left(l\right)}$$

The magnesium dissolution from the substrate and the consequent deposition of magnesium fluoride on the surface occurred with a dynamic balance. The reaction (5), occurring at substrate-medium interface, was gradually decreased by the fluoride formation; in fact, MgF_2_ was not soluble in water and acted as a diffusion barrier to ions migration. After an initial growth, MgF_2_ deposition was slowed down by the increasing thickness. After 12 h of immersion in HF, the barrier film was thick enough to terminate the reaction. The small pores detected on the coating surface could be caused by the H_2(g)_ evolution that accompanied the formation of MgF_2_.

### 3.2. Electrochemical Results

**Figure 9:**
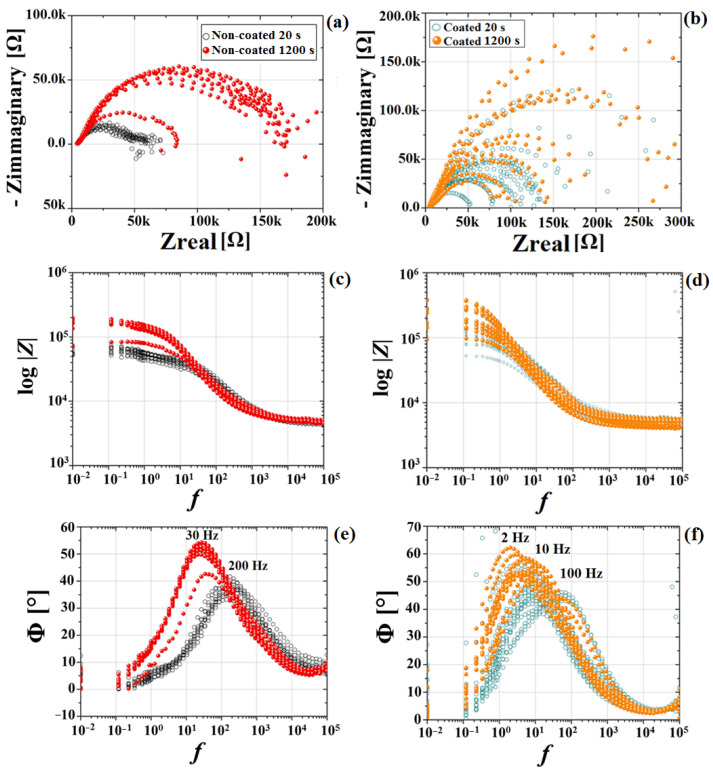
EIS spectra obtained for non-coated (left) and coated (right) samples at the start (20 s) and after 1200 s exposure to the electrolyte. Nyquist plot (**a**,**b**); Bode modulus (**c**,**d**); Bode phase (**e**,**f**).

The time-dependent corrosion behavior of non-coated and MgF_2_-coated Mg-alloys was evaluated and compared in terms of short exposure times. Nyquist and Bode plots after 20 and 1200 s of OCP are reported in Figure 9.

The uncoated samples exhibited only one capacitive loop, which represented the interface reaction between the electrolytic solution and the substrate, as already reported for Mg [62,63]. The phase angle had a peak at 200 Hz, which shifted over time until 30 Hz after 1200 s exposure to the electrolyte with a phase angle increase (Figure 9e). This behavior was attributed to the formation of an oxide layer on the sample surface and the slowing down of the corrosion process. For coated samples, the EIS plots consisted of two capacitive loops mostly overlapping, but with two distinctive characteristic frequencies in the in high (10^3^ Hz) and medium (10 Hz) range frequency regions, indicating that two parallel processes occurred at the surface (Figure 9f). The high frequency capacitive loop represented the behavior of the coating-electrolyte interface, while the medium-range frequency capacitive loop was descriptive of the coating-substrate interface. Based on the extrapolation of the Nyquist plots, the values of polarization resistance (R_p_), directly related to the corrosion resistance of the material, were obtained (Table 3). Initially, the R_p_ values of the coated samples were higher than those of the uncoated samples, confirming a significant decrease in the initial degradation rate due to the MgF_2_ layer, so that an improvement of the overall corrosion resistance of the studied magnesium alloy was registered.

**Figure 10:**
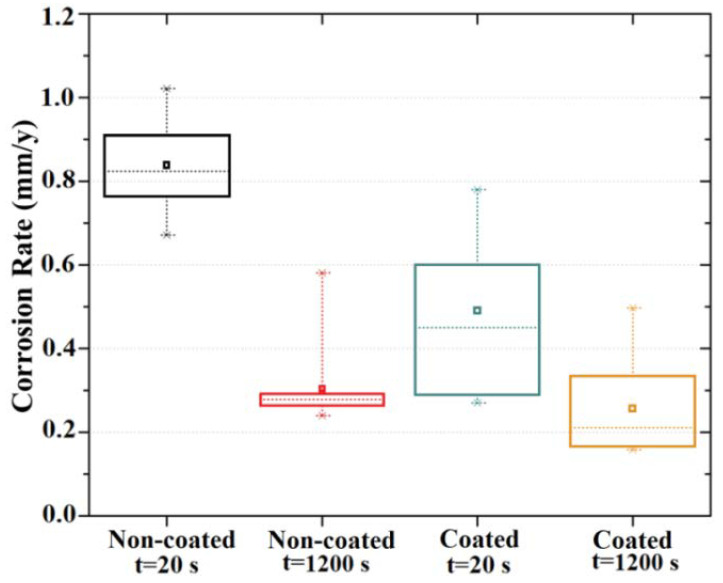
Box plot of the corrosion rate results obtained for non-coated and MgF_2_-coated samples.

The initial average corrosion rate of the coated samples, when compared to the uncoated alloy, decreased from 0.84 mm/y to 0.49 mm/y, as observed from the EIS results. Additionally, the coated samples did not show a significant statistical variation in polarization resistance between 20 s and 1200 s when in contact with the electrolyte (Figure 10).

**Figure 11:**
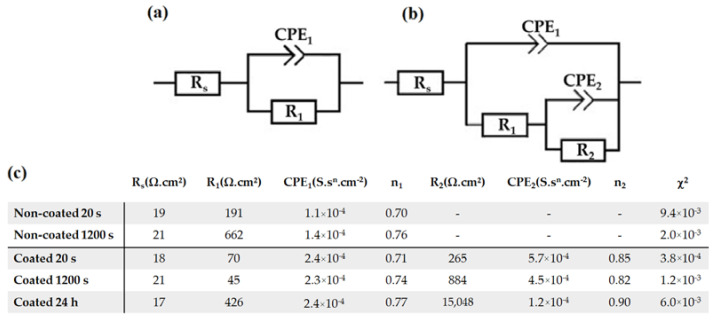
Electrical equivalent circuits used in this study. Representation of equivalent circuit for non-coated (**a**) and coated (**b**) samples, respectively, and the fitted electrical parameters (**c**).

In contrast, uncoated samples showed a considerable Rp variation when in contact with the electrolyte. This was also evident from the Bode phase graph, which showed a phase angle shift from 40 to 60° after 1200 s of exposure (Figure 9e), indicating an improvement in terms of corrosion resistance. This phenomenon, already observed and reported by Witte et al. and by Jamesh et al. [64,65], was related to the thickening of the corrosion product layer (usually Mg(OH)_2_), which acted as an effective barrier for the charge transfer process. The fitting of the EIS data using RC equivalent circuits could be satisfactorily achieved using the equivalent circuits represented by (Figure 11a,b), composed by R_s_, the electrolyte resistance, R_1_ and CPE_1_, representing the surface resistance and capacitance of the double layer, respectively. The additional (R_2_ and CPE_2_) circuit components stand for the surface resistance and capacitance of the innermost part of the MgF_2_ coating [66,67]. Therefore, the Rp for the coated samples is represented by R_1_+R_2_. A constant phase element (CPE) was used instead of a pure capacitor. The CPE behavior is a form of distortion from an ideal electrical capacitor. It can be explained by local changes in the electrical field and in the potential of the surface, arising from surface heterogeneities and roughness [68]. The fitted electrical parameters, including the exponent “n” that indicates the deviation of the CPE from the ideal capacitive behavior, are shown in Figure 11c, and have been documented for magnesium alloys [50]. Analysis of the data shows that the film resistance (R_1_+R_2_) increased with exposure time, in particular for the samples coated with MgF_2_. Moreover, these results confirm that the film formed on the surface of Mg in buffered environments plays a protecting role for the substrate, as reported in [69]. Compared with this naturally formed film, the compactness and thickness of the MgF_2_ coating acts as a barrier against the corrosion process of Mg samples, significantly increasing the impedance, as shown in [41].

**Figure 12:**
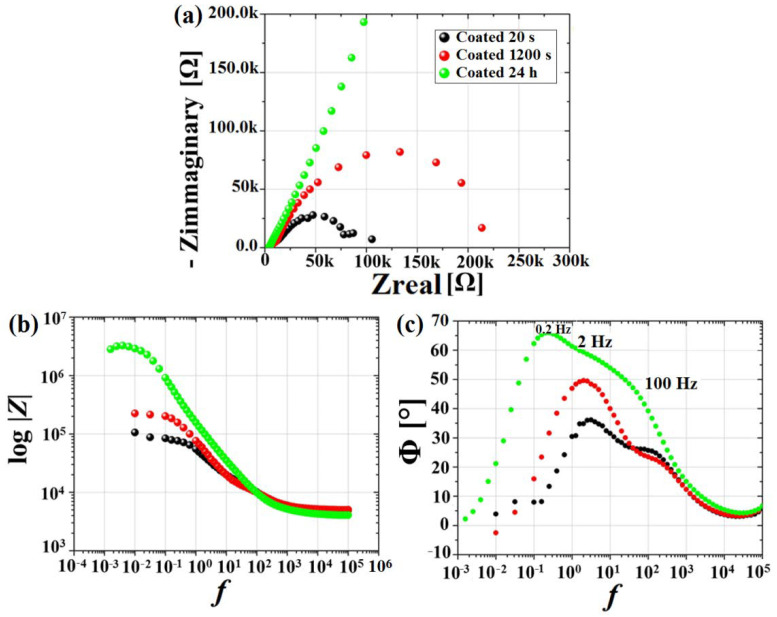
EIS spectra obtained for coated samples at the start (20 s) and after 1200 s and 24 h of exposure to the electrolyte. Nyquist plot (**a**); Bode modulus (**b**); Bode phase (**c**).

These results were further confirmed by the prolonged exposure test of the coated samples over a period of 24 h, as shown in Figure 12. The two capacitive arcs, also found for short exposure times, became more pronounced (Figure 12c). Moreover, there was a significant increase in the capacitive arc in the Nyquist plot over time, with the corrosion rate decreasing from 0.49 mm/y at the beginning of the experiment, to 0.01 mm/y after 24 h. This impedance increase could be related to the presence of corrosion products filling the micro-cavities through the coating and along the grain boundaries. The formation of corrosion products acted as a barrier between the electrolyte solution and the coated magnesium, resulting in the Rp growth and, consequently, a significant decrease of the corrosion rate, as reported in [49].

## 4. Conclusions

NOVAMag^®^ fixation screws were characterized before and after a fluoride conversion treatment, and applied to the bare alloy with the intent to reduce the corrosion rate in the first hours of implantation in human bones. The following conclusions were determined:

(1) A successful formation of a mixed MgF_2_/MgO coating, about 2 µm thick, after immersion in HF, was obtained.
(2) The topography of the coated screws, being smoother than that of the non-coated screws, showed homogenous features, exhibiting the formation of grooves with a more similar dimension and were evenly distributed.
(3) The presence of both metal-bound-F and metal-bound-O at the uppermost nm of the coated samples surfaces were detected.
(4) The MgF_2_ coating demonstrated an efficient role in retarding alloy degradation during the initial stages of exposure to the chloride-containing physiological solution up to 24 h, with the corrosion rate determined using EIS spectra decreasing from 0.49 mm/y to 0.01 mm/y.

These results demonstrated the effectiveness of the applied MgF_2_ film in slowing down alloy corrosion, allowing these materials to be competitive as dental and orthopedic products in the biodegradable implants market. A mechanical characterization, including nano-scratch-tests, is suggested to further investigate micro-hardness and coating adhesion and cohesion, respectively, in order to predict screw performances in an application scenario. For this purpose, a special set-up capable of characterizing these kinds of samples with complex shapes and small dimensions is required, and is currently under study.

## Figures and Tables

**Table 1:** Recent studies on MgF_2_ coating on Mg-based materials.

Mg-Alloy	Sample Shape	Treatment	Outcomes	Applications	Ref.
Mg-3Zn-0.5Zr	Cylinders (Ø = 3 mm; h = 10 mm)	HF (20%); 6 h; 37 °C	Decreased corrosion rate; promotion of new bone formation	Orthopedic applications	2017 [38]
Mg-2Zn-0.5Y-0.5Nd	Discs (Ø = 10 mm; h = 2 mm)	HF (40%); 24 h; room temperature	No subcutaneous gas cavities or significant inflammatory cell infiltration	Cardiovascular stents	2019 [39]
AZ31	Plates (5 mm × 7 mm × 2 mm)	Microfluoruration in saturated NH_4_HF_2_ solution at 190 V	Improved corrosion resistance; reduced H_2(g)_ bubbles generation	Medical implant materials	2021 [40]
Mg (99.98%)	Sheets (12 mm × 10 mm × 1 mm)	HF (40%); 24, 48, 96 h; room temperature	Decreased corrosion rate; great cytocompatibility; good attachment and growth of osteoblasts on the surface	Orthopedic applications	2021 [41]
Mg (>99.99 wt.%)	Plates (10 mm × 1 mm × 2 mm) Rodes (Ø = 10 mm; h = 2 mm)	HF (40%); 72 h; room temperature	Beneficial for the osteogenic differentiation of MC3T3-E1 cells and the vascularization of human umbilical vein endothelial cells (HUVECs)	Orthopedic applications	2020 [42]
Mg-2Y-1Mn-1Zn	Screws	HF (40%); room temperature	Decreased corrosion rate; reduced H_2(g)_ bubbles generation	Orthopedic applications; Bone regeneration	This work

**Table 2:** Nominal composition of WZM211 alloy (wt.%).

	Y	Zn	Mn	Mg
WZM211	2.0	1.0	1.0	bal.

**Table 3:** Average polarization resistance and corrosion rates obtained for the machined and coated samples tested.

	Non-Coated	Coated	Non-Coated	Coated
	Rp [Ω.cm^2^]		CR [mm/y]	
20 s	359 ± 14	694 ± 85	0.84 ± 0.03	0.49 ± 0.06
1200 s	1036 ± 198	1323 ± 144	0.30 ± 0.03	0.26 ± 0.03

## Data Availability

The data presented in this study are available on request from the corresponding author(s). The data are not publicly available due to the fact that they are a subset of a bigger dataset covered by industrial secret (*biotrics implants AG*).
